# Inferring functional transcription factor-gene binding pairs by integrating transcription factor binding data with transcription factor knockout data

**DOI:** 10.1186/1752-0509-7-S6-S13

**Published:** 2013-12-13

**Authors:** Tzu-Hsien Yang, Wei-Sheng Wu

**Affiliations:** 1 Department of Electrical Engineering, National Cheng Kung University, Tainan City, Taiwan (R.O.C

**Keywords:** functional TF-gene binding pair, transcription factor knockout data, chromatin immunoprecipitation

## Abstract

**Background:**

Chromatin immunoprecipitation (ChIP) experiments are now the most comprehensive experimental approaches for mapping the binding of transcription factors (TFs) to their target genes. However, ChIP data alone is insufficient for identifying functional binding target genes of TFs for two reasons. First, there is an inherent high false positive/negative rate in ChIP-chip or ChIP-seq experiments. Second, binding signals in the ChIP data do not necessarily imply functionality.

**Methods:**

It is known that ChIP-chip data and TF knockout (TFKO) data reveal complementary information on gene regulation. While ChIP-chip data can provide TF-gene binding pairs, TFKO data can provide TF-gene regulation pairs. Therefore, we propose a novel network approach for identifying functional TF-gene binding pairs by integrating the ChIP-chip data with the TFKO data. In our method, a TF-gene binding pair from the ChIP-chip data is regarded to be functional if it also has high confident curated TFKO TF-gene regulatory relation or deduced hypostatic TF-gene regulatory relation.

**Results and conclusions:**

We first validated our method on a gathered ground truth set. Then we applied our method to the ChIP-chip data to identify functional TF-gene binding pairs. The biological significance of our identified functional TF-gene binding pairs was shown by assessing their functional enrichment, the prevalence of protein-protein interaction, and expression coherence. Our results outperformed the results of three existing methods across all measures. And our identified functional targets of TFs also showed statistical significance over the randomly assigned TF-gene pairs. We also showed that our method is dataset independent and can apply to ChIP-seq data and the *E. coli *genome. Finally, we provided an example showing the biological applicability of our notion.

## Background

Cellular responses to external stimuli or environmental changes are usually conveyed through cellular regulatory networks consisting of different regulatory pathways [1-4]. Transcriptional regulation plays an essential role for construction of such regulatory pathways at the level of transcription. The binding of specific transcription factors (TFs) controls the initialization or the expression level of genes. Thus, unravelling functional TF-gene binding events is a fundamental start-up for us to understand the regulatory mechanisms in cells [1].

Chromatin immunoprecipitation experiments (ChIP-chip or ChIP-seq) are now the most comprehensive experimental approaches for mapping the binding of TFs to their target genes [2,3,5]. However, ChIP data alone are insufficient for identifying functional binding target genes of TFs for two reasons. First, there is an inherent high false positive/negative rate in ChIP-chip or ChIP-seq experiments [6]. Although by controlling the level of statistical significance for the analysis can reduce the false positive rate, this approach is prone to getting a great number of false negatives [7,8]. Second, binding signals in the ChIP-chip data do not necessarily imply functionality. The binding of TFs to the promoters of genes may not lead to subsequent transcription activation/repression [9,10].

It was suggested that one can improve the confidence of the TF-gene binding pairs by integrating ChIP-chip data with data from other high-throughput technologies [10]. Although other high-throughput data may themselves be noisy, the stochastic noises are generally assumed to be uncorrelated [9-11]. Hence, combining different sources of high-throughput data is a promising way of extracting biologically meaningful information embedded in any noisy high-throughput data.

Previous studies had tried to extract functional binding target genes of TFs by integrating the ChIP-chip data with various kinds of high-throughput data. By the types of the integrated data, the integration processes could be roughly divided into two categories. The first type of existing methods relied on stepwise integration of the ChIP-chip data with the expression data and/or the TF binding motif data. Functionality of the TF-gene binding pairs was confirmed by some gene properties inferred from the mRNA expression profiles. For example, ChIP positives were classified into functional and non-functional TF-gene binding pairs by the regression analysis of the mRNA expression profiles [12]. And others tried to infer functional binding target genes of TFs from the ChIP-chip data by the synergy properties derived from the mRNA expression profiles and the TF binding motif data [10]. Finally, another group of researchers developed the CERMT algorithm to refine the possible functional binding target genes of TFs based on covariance of multiple expression time series [13].

The other type of existing bioinformatic approaches for extracting functional TF-gene binding pairs combined diverse biological data beside the mRNA expression profile data through the construction of different types of Bayesian classifiers. Some utilized the framework of probabilistic inference to predict the functional TF-gene binding pairs by TF binding site motifs, evolutionary conservation, regulatory potential, nucleosome data and DNase hypersensitive sites [14]. Others constructed a Bayesian classifier from comprehensive sources of yeast high-throughput data such as protein-protein interaction data, the phylogenetic data and the nucleosome data [7,8]. Another group specified a hierarchical Bayesian model to augment the protein-DNA binding data with gene expression and sequence data [15]. Still others defined and trained a logistic regression classifier based on a mapping of preference scores on gene location information and TF-binding motifs [9].

While previous works had combined comprehensive sorts of high-throughput experimental data and biological data, these approaches did not consider the TF-gene regulatory relation when inferring functional TF-gene binding pairs. Expression data, TF binding motif data and other integrated biological data, such as nucleosome positioning and evolutionary conservation, did not directly provide the TF-gene regulatory relation. Nowadays, the TF knockout (TFKO) data are available for biologists to infer the TF-gene regulatory relation [4]. TFKO data convey the experimental results showing the change in the expression of some target gene caused by the deletion or mutation of certain TF-encoding gene, revealing the fact that the TF regulates this target gene via certain mechanisms [16]. Since none of previous methods had directly utilized the TF-gene regulatory relation, we propose an alternative to infer functional TF-gene binding pairs based on the integration of ChIP-chip data with TF-gene regulatory relation.

In this study, instead of using the supervised or unsupervised learning tools as in the Bayesian approach and other methodologies, our method uses a network approach on the combination of the ChIP-chip data and the TFKO data to infer the functional TF-gene binding pairs. A TF-gene binding pair, or a ChIP positive, is called functional if we can also find evidence showing that the TF regulates the expression of the target gene. While direct overlapping of the ChIP-chip and TFKO datasets could give some possible functional TF-gene binding pairs, this only provided a very small number of such pairs because of the low overlap of the ChIP-chip data and the TFKO data [4,17,18]. It was shown that the low overlap between the TFKO data and the binding data partly resulted from knockout epistasis [4] or backup mechanisms [17]. The epistatic regulation cascade from the given TF-pair with a higher confident regulation of an intermediate TF on the target gene is suggested to compensate the knockout effect of the regulation of this hypostatic TF-gene pair. Hence we further considered the possible hypostatically masked (to the epistatic regulation cascade) TF-gene regulation relation deduced from the original TFKO data. The literature-curated TFKO regulation relation and the deduced hypostatic regulation regulation for given ChIP positive TF-gene binding pairs were also checked through regulatory confidence scores (RCS). Finally, a TF-gene binding pair with a confident TF-gene regulation, which may be the curated TFKO regulation or deduced hypostatic TF-gene regulation, was classified to be functional. We validated the proposed method on a gathered ground truth set and also demonstrated the superior biological significance of our method to three previous methods by testing the results on functional enrichment, the prevalence of protein-protein interaction and target gene co-expression. Of all three different aspects of biological significance demonstration, our results all showed improvement over the three previous works. We also showed that our method is dataset independent and can apply to ChIP-seq data and the *E. coli *genome. Finally, we provided an example showing the biological applicability of our notion.

## Materials and methods

### ChIP-chip data and TF knockout data

Genome-wide in vivo TF-gene binding data of 204 yeast *Saccharomyces cerevisiae *TFs produced by the ChIP-chip technology were adopted from [3]. The TF-gene binding assignments were provided in the form of binding *p*-values, on the hypothesis that the TF binds to the promoter region of the target gene. To show the data-independence of our method, we also adopted the ChIP-chip data generated from [2]. In their location analysis protocol, a promoter region of a gene is defined as the upstream intergenic region. The genome wide intergenic regions were obtained and amplified using the Yeast Intergenic Region Primers (Research Genetics) [19]. In *Saccaromyces cerevisiae*, transcription factor binding sites are positioned further upstream in the intergenic regions and vary over a wide range in promoters [20]. In this study we adopted the promoter definition and promoter regulation as the ones used in the study of Harbison *et al. *[3].

The TF knockout data of 156 yeast *Saccharomyces cerevisiae *TFs were retrieved from the Yeastract Indirect evidence [16]. Yeastract has deposited the published data showing the change in the expression of the target genes resulting from the deletion or mutation of certain TF-encoding genes. This so-called indirect evidence therefore provides the TF-gene regulation information. We retrieved 21871 TFKO TF-gene regulation pairs for 156 TFs from Yeastract.

### Protein-protein interaction data and mRNA expression data

Two different datasets were collected for use in the biological validations. For showing the prevalence of protein-protein interaction, we gathered the physical protein-protein interaction data from the Biogrid database, which had deposited comprehensive collections of protein-protein interactions [21]. And for comparing the expression coherence between different methods, we retrieved 40 time series mRNA expression profiles in yeast *Saccharomyces cerevisiae *from ExpressDB [22]. These 40 different expression conditions were obtained as previously suggested [10]. Details of the 40 different conditions can be found in the online supplementary files of [10]. These conditions represent the natural and perturbed expression profiles, including the conditions under sporulation in budding yeasts [23], yeast cell cycle conditions [24,25], the DNA damaged conditions [26,27] and etc.

### Benchmark control sets

A set of 484 functional TF-gene binding pairs adopted from [7] were used as the positive control set. These literature-curated ground truth functional TF-gene binding pairs were collected from the Incyte YPD Database. To obtain the negative control set, we generated 1516 random TF-gene pairs. To enhance the stringency of the negative control set, we further required the random pairs not to belong to the positive control and not to have any literature evidence curated in the Yeastract database [16]. A total of 2000 TF-gene pairs were used as the control set.

### Finding the hypostatic TF-gene regulation relation

We used the literature-curated TF-gene regulation pairs from the TFKO data to construct a regulatory relation network. An edge from a given TF to its regulatory target gene was added to the regulatory relation network if there is TF-gene regulatory relation from the TFKO data showing that the TF regulates the target gene. For a given TF-gene binding pair, if they are connected by a path of length of two with an intermediate node TF *X *in the constructed regulatory relation network, this means that the given TF regulates the TF *X *and the TF *X *regulates the given gene. We said that there is deduced hypostatic regulatory relation (to the epistatic regulation cascade through TF *X *on the target gene) in the constructed regulatory relation network for this given TF-gene pair (Case II in Figure 2). And the knockout effect of this hypostatic regulation relation may thereby be masked. Epistatic regulation cascade path of length more than two can be inferred in a similar manner.

We searched such deduced hypostatic regulatory relation of a TF-gene binding pair by the modified breadth first search (mBFS) algorithm [18]. The algorithm returned the shortest regulation path between a given TF-gene pair in the regulatory relation network. To briefly explain the algorithm, two different sets of nodes were kept, one for the visited nodes and one for the discovered nodes. First, we started out from the given TF and put it in the set of visited nodes. Then we tried out all of its "unvisited" neighbours in the regulatory relation network and put the neighbours in the set of discovered nodes. This process was repeated for each node in the set of discovered nodes in the "first-in, first-out" manner, acting as a new starting node in each round, until we reached the target gene. The shortest regulation path could be obtained by tracing back the process.

### Calculating the RCSs for the confidence of the TF-gene regulation

The deduced hypostatic TF-gene regulatory relation might be introduced by chance since there is still a large amount of random noises in the original TFKO data due to the inherent uncertainty in high-throughput technologies. These inherent random noises could cause the over-fitting problem when deducing hypostatic relations from analysing the network paths [17]. We avoided the stochastically introduced TFKO regulation or epistatic relation cascade by comparing the paths found in the constructed regulatory regulation network with those found in the randomly generated network. We forced the random networks to preserve the node degrees to mimic the degree distribution of the original TFKO regulatory relation pairs [28]. Then we used the Student *t*-test to test against the null hypothesis that the length of the shortest regulation cascade found in the constructed regulation relation network is statistically equal to the average of the lengths of the shortest paths in the randomly generated regulation network. Multiple hypotheses test correction was done by using the method of FDR. And the regulation confidence score (RCS) is calculated by the formula, which takes the minus logarithm on the corrected statistic *p*-value:

RCS=-log(p_value)

The RCS measures the non-stochastic confidence of the given regulation pair.

To calculate RCSs, we constructed 10000 degree-preserving TFKO random regulation networks. The choice for sampling size of 10000 from the random distribution is to have a sampling precision of 95% confidence within 1% of error, according to the sampling theorem [29]. To generate the degree preserving random network, first the degree sequences for nodes in the regulatory relation network were generated, including both the in-degree sequence and the out-degree sequence. Then we expanded the degree sequences into node frequency sequences. For example, if we have an in-degree sequence of {3,2,1}, then we get an in-degree node frequency sequence of {1,1,1,2,2,3} with respect to the given in-degree sequence, for the first node having three in-coming edges in the network. Then we randomly shuffled the in-degree node frequency sequence and the out-degree node frequency sequence. Edges in the random network were added for nodes from the randomly shuffled out-degree node frequency sequence to nodes from the randomly shuffled in-degree node frequency sequence iteratively. This guarantees the random networks with the degree preserving property [30]. Details of the degree distribution and the properties of the random networks can be found in [30].

## Results and discussion

### Overview of the approach

**Figure 1:**
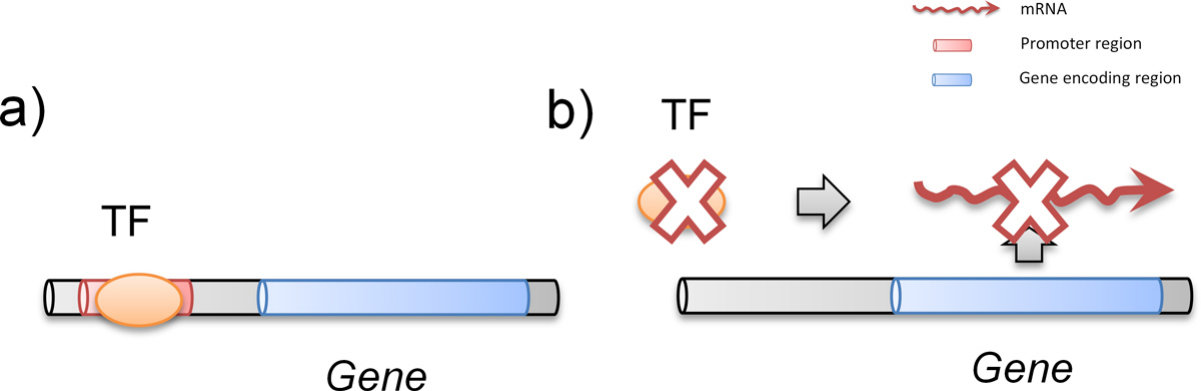
**Properties of ChIP data and TFKO data**. a) ChIP-chip data shows that a TF directly or indirectly binds to the promoter region of the target gene. b) TFKO data shows the change in the expression of some target gene resulting from the deletion or mutation of certain TF-encoding gene.

**Figure 2:**
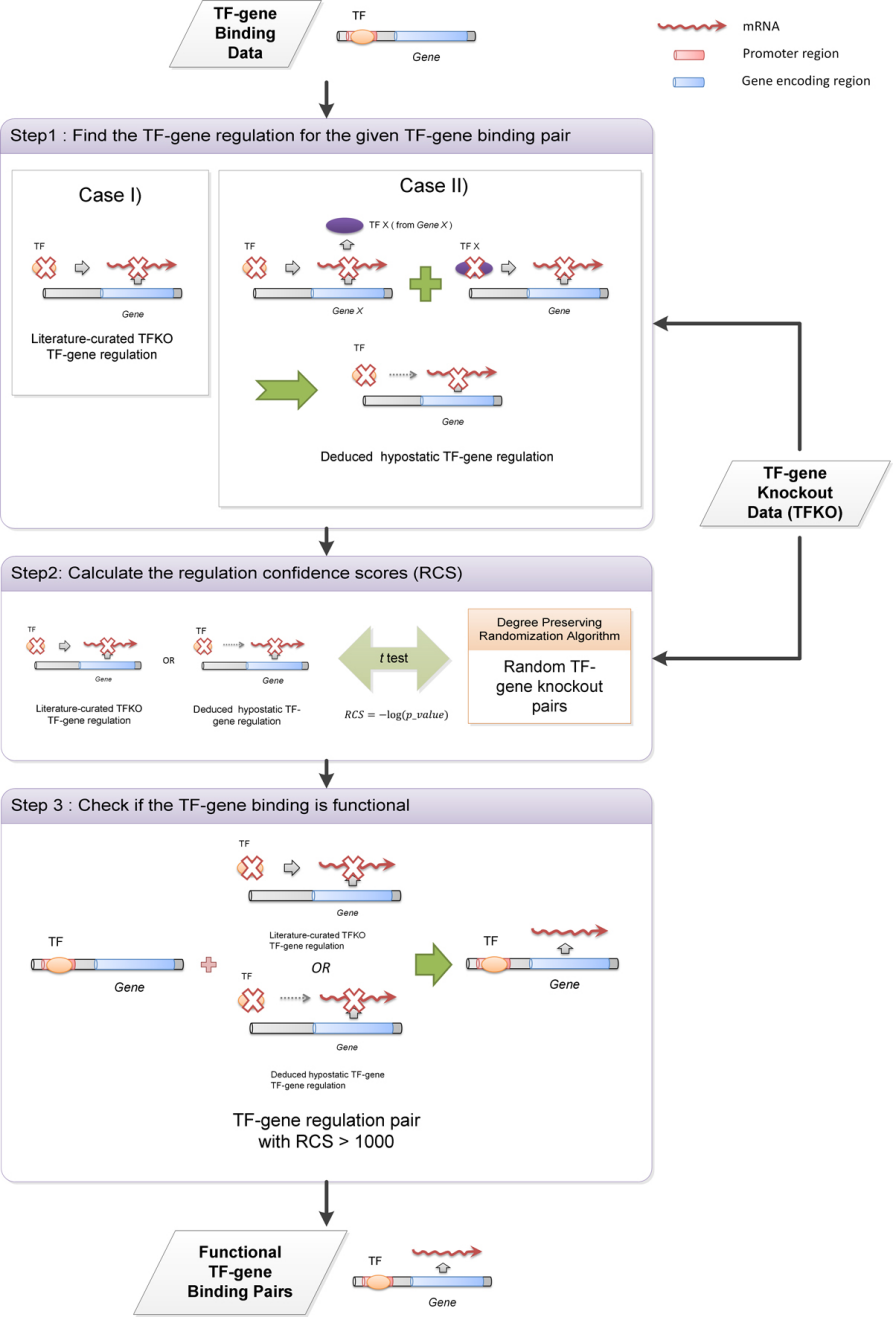
**The algorithmic scheme to extract functional TF-gene binding pairs**. We used a network approach to combine the ChIP-chip data and the TFKO data. The central notion of our method is based on the deduced hypostatic TF-gene regulation. This extended the low overlap between the binding data and the TFKO data. For a given TF-gene binding pair, we first sought for regulation evidence for this pair. The regulation evidence can be either the direct literature-curated TFKO TF-gene regulation or the deduced hypostatic TF-gene regulation. Then we calculated the confidence for the TF-gene regulation by comparing the regulation pair with the same pairs in randomly generated TFKO networks. A final RCS was calculated for confidence measurement. A TF-gene binding pair is called functional if it also has confident TF-gene regulation information (with RCS > 1000).

TFKO data conveys the experimental results showing the change in the expression of some target gene resulting from the deletion or mutation of certain TF-encoding gene and the ChIP-chip data conveys the TF-gene binding information (Figure 1). To extract the functional TF-gene binding pairs, we used a network approach to combine the ChIP-chip data and the TFKO data. The overall algorithm is depicted in Figure 2. We started out from the ChIP positives as the potential functional TF-gene binding pairs. As mentioned, a ChIP positive is called functional if we can also find evidence showing that the TF regulates the expression of the target gene. Hence first we sought two different possible TF-gene regulation relation from the TFKO data: the curated TFKO TF-gene regulation (Case I in Figure 2) and the deduced hypostatic TF-gene regulation (Case II in Figure 2).

For a given TF-gene binding pair, if there was no literature-curated TFKO TF-gene regulation for it, we then tried to see if there exists a possible hypostatic TF-gene regulation for it. It was shown that the low overlap between the binding data and the TFKO data may partly result from knockout epistatic mechanisms and a single TF knockeout effect on a target gene may be compensated by the epistasis regulation cascade through another paralogous partner TF *X *[4,17] (Case II in Figure 2). Note that TF *X *may not directly bind the target gene. This innovated us to find the possible masked hypostatic TF-gene regulation. The compensated TF-gene regulation was said to be hypostatic to the epistatic regulation cascade through TF *X *since the knockout effect of this TF-gene pair may possibly be masked by the epistatic regulation cascade with a more confident regulation of an intermediate TF *X *on the given target gene. This meant that there might exist at least an epistatic regulation cascade, or a path from the TF to its target gene through an intermediate TF *X *in the regulatory relation network, for this TF-gene pair. Therefore, we constructed a regulatory relation network from the TFKO data and sought the hypostatic TF-gene regulation relation by checking the existence of a regulation path in the regulation relation network for the given ChIP postives. This was done by a previously published path finding algorithm (as described in the Methods section).

Since there are inherent uncertainties in the high-throughput technologies, the TFKO regulation relation or the deduced hypostatic TF-gene cascade regulation may be introduced by chance. Because of this reason, as a second step, the curated TFKO regulation relation or the deduced hypostatic regulation relation for the given TF-gene ChIP positive was also checked by the regulatory confidence score (RCS), which was scored through the comparison with random TFKO data (See Methods section). A regulation relation with RCS higher than 1000 was set to be confident. Finally, a TF-gene binding candidate pair was classified to be functional if it has a confident TF-gene regulation evidence, which may come from the curated TF-gene regulation or the hypostatic TF-gene regulation.

### Validation on a literature-proven benchmark TF-gene set

**Figure 3:**
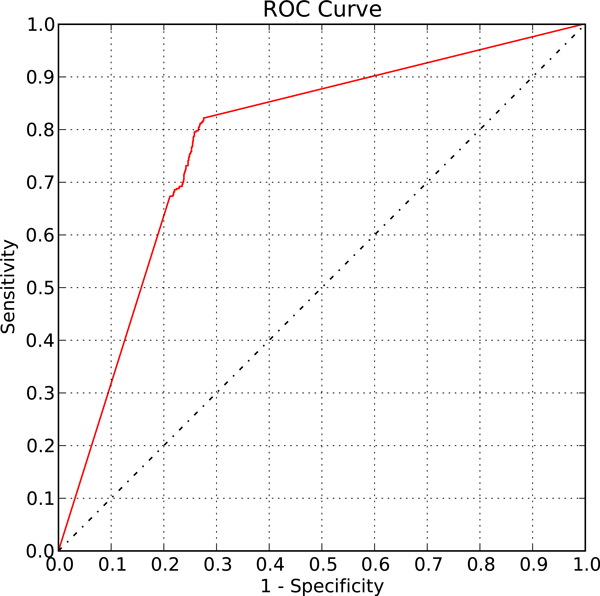
**The ROC curve of the validation on the ground truth set for Saccharomyces cerevisiae**. When applied to the control set for *S. cerevisiae*, our method can distinguish functional binding targets from non-functional binding pairs, shown by the upper left corner trend on the ROC curve plot with AUC= 0.78. The ground truth set consists of 484 literature-curated functional TF-gene binding pairs as the positive control set and 1516 random TF-gene pairs as the negative control set.

First we validated our proposed method on a gathered literature-curated functional TF-gene binding set from [7]. The literature-proven functional TF-gene binding pairs were treated as the positive control set and the randomly generated TF-gene pairs were viewed as the negative control set. Applying our method on the prepared control set, we can generate the receiver operating characteristic (ROC) curve by adjusting the regulatory confidence scores (RCSs) (Figure 3). The RCS is a measurement for the confidence of the curated TFKO TF-gene regulation relation or the deduced hypostatic TF-gene regulation relation as described in the Methods section.

An ROC curve plot is a graphical tool demonstrating the performance of the discriminating algorithm as its discriminating score varies. The curve is plotted for (1-specificity) against sensitivity. Specificity is defined as the fraction of true negatives out of the discriminated negatives and (1-specificity) is also known as the false positive rate. And sensitivity, also known as the true positive rate, is defined as the fraction of true positives out of the discriminated positives. In the ROC curve plot of our method, we can see that our method acted as a good classifier for discriminating functional binding pairs from non-functional binding pairs (area under curve, AUC = 0.78) due to its performance of low false positive rates with high true positive rates (to the leftmost of the ROC curve plot). Notice that the trembling phenomenon between 0.2 and 0.3 shows that most of the discriminating scores, which is the RCS, resulted in false positive rate of 0.2 to 0.3 with true positive rate of about 0.7 to 0.8. Since our method does not rely on any training process, this result is unlikely to suffer from over-fitting. Hence we conclude that our method can distinguish functional TF-gene binding pairs from non-functional binding pairs.

### 82% of the original TF-gene binding pairs suggests functionality

In this study, we demonstrated our algorithm on yeast *Saccharomyces cerevisiae *because of the comprehensibility and availability of the genome-wide TFKO data source. Harbison *et al. *have performed the most comprehensive genome-wide chromatin immunoprecipitation microarray (ChIP-chip) experiments on the yeast *Saccharomyces cerevisiae *[3]. And from the experimental results, they reported the binding target genes of 204 TFs. It was suggested taking a *p*-value threshold of 0.001 in the original error models to ensure a low false positive rate. But it has been shown that the TF-gene binding pair might already be functional with the *p*-value threshold of 0.01 [7]. Hence in this study, we took the threshold of 0.01 to get start-up ChIP positives for identifying functional binding targets.

**Figure 4:**
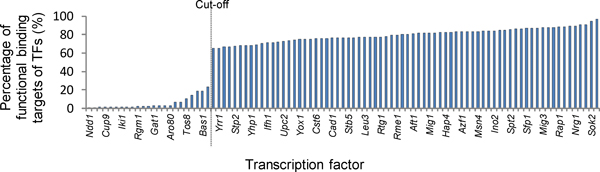
**The percentage distribution of functional binding targets of TFs extracted from the original ChIP-chip dataset**. The potential binding target genes were defined by a *p*-value threshold of 0.01. As the bar chart shows, there is a 'jump' from 23% to 65% of extracted functional binding target genes. Hence, a cut-off that at least 25% of the original binding target genes should be functional was adopted. This gives 72 analysable TFs with their functional binding target genes (ratio of functional binding targets for these 72 analysable TFs: mean = 79.68%, standard deviation = 7.3%). Only parts of the TF names were marked in the plot due to space limitations.

Only a subset (95 TFs) of the reported TFs in the study of Harbison *et al. *were possible for our analysis because of the lack of the TFKO data. After applying our proposed algorithm, we further required that the percentage of the functional binding targets of a TF should reach above 25% since we observed a 'jump' from 23% to 60% in the percentage distribution of the extracted functional binding target genes (Figure 4 and Additional File 1). Since the binding pairs adopted from Harbison *et al. *have been already restricted to the TF-gene binding pairs that fit into the promoter binding model, this 'jump' indicated that the low percentage of functional binding targets of certain TFs might also result from the lack of TFKO data. As a result, there were 72 TFs suitable for our analysis and a total of 7259 functional TF-gene binding pairs were established by our method (See Additional File 2). On average, there are about 82% (7259/8904) functional TF-gene binding pairs in the original ChIP-chip data for the 72 analysable TFs. Direct overlapping of the ChIP-chip data and the TFKO data resulted in 1220 functional TF-gene pairs. And we have expanded the number of functional TF-gene binding pairs by about 6 folds. We used these 72 analysable TFs with percentages of the functional binding targets above 25% in the following validation.

### Biological significance comparison with previous methods

We next compared the biological significance of the functional TF-gene binding pairs identified in this study and by three previously published methods. Only three approaches on yeast *Saccharomyces cerevisiae *were selected for our comparison because of data availability. For the methods of combining diverse biological data sources to extract the functional TF-gene binding pairs, the log likelihood score (LLS) method [7] is available for our comparison. The LLS method integrated the most comprehensive experimental datasets to train the Bayesian classifier, where ChIP-chip data, TF binding motif data, data of sensu stricto species of *Saccharomyces cerevisiae*, co-expression clustering, physical protein-protein interaction data and the phylogenetic profiles of gene pairs were used.

For the methods relying on stepwise integration of the ChIP-chip data with the expression data, there are two approaches available for our comparison. One is the method of using the expression coherence score (ECS) and TF binding sites information [10] and the other is the method of MA-networker (MA) algorithm [12]. The ECS method was based on the integration of co-expression clustering, TF binding motifs, TF synergistic interactions and the TF co-localization in the promoter regions of target genes, which were mostly evaluated by the EC scores. And the MA-networker algorithm classified the ChIP-positives into functional and non-functional targets based on their expression patterns across different experimental conditions and the transcription factor occupancy data.

In the following sub-sections, we showed that our results conveyed better biological relevance than these three previous works by testing the identified functional binding target genes of TFs on functional enrichment, the prevalence of protein-protein interaction and co-expression. Details of the following validations can be found in Additional File 3.

#### Functional enrichment analysis

When several genes are functionally bound by the same TF, one might expect that the gene products of these genes are prone to carry similar cellular functions [7,31]. Gene ontology (GO) terms provide this sort of characterization. Following the definition of [7], the target gene set of a TF is called functionally enriched if the gene set significantly overlaps with at least one gene ontology category across the three different GO categories (the biological process ontology, the molecular function ontology and the cellular component ontology). Based on this notion, the functional enrichment test was performed by the web-based tool, GO Term Finder [32]. The statistical functional GO term enrichment test was implemented by one-tailed Fisher Exact Test in Go Term Finder. The statistical results then went through FDR correction for multiple hypotheses tests. For our analysis, we took a *p*-value threshold of 0.05.

**Figure 5:**
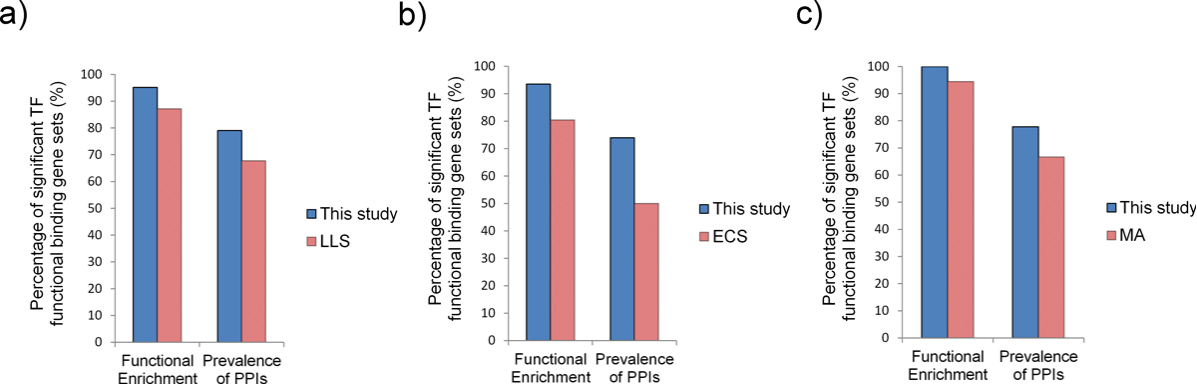
**Functional enrichment and prevalence of protein-protein interaction validation**. Functional binding target gene sets were validated by their functional enrichment and prevalence of protein-protein interaction (PPI). Note that the total numbers of TFs in each chart differ and correspond to the number of common TFs between those available from this study and from the method under comparison. a) Our results compared with results of the LLS method (62 common TFs). b) Our results compared with results of the ECS method (46 common TFs). c) Our results compared with results of the MA method (18 common TFs).

Of the 62 common TFs between our results and the results of the LLS method, 59 TF functional binding target gene sets (95.2%) extracted by our method showed significant functional enrichment while only 54 TF functional binding target gene sets (87.1%) extracted by the LLS method bore significant functional enrichment. And comparing the 46 common TFs between our results and the results of the ECS method, our results still outperformed the results of the ECS method (43 functionally enriched TF functional binding target gene sets compared with 37 functionally enriched TF functional binding target gene sets, i.e. 93.5% compared with 80.4%). As for the 18 common TFs between our results and the results of the MA algorithm, our results showed better functional enrichment (18 functionally enriched TF functional binding target gene sets compared with 17 functionally enriched TF functional binding target gene sets, i.e. 100% compared with 95.6%) (Figure 5). Note that the high percentage of 100% achieved in the comparison to the results of MA algorithm is mainly due to the scare available common functional gene target sets of TFs between our results and that of MA algorithm. In summary, our method can extract functional binding target genes of TFs with better functional enrichment than previous approaches.

#### Prevalence of protein-protein interaction

Functionally related genes tend to carry similar cellular functions by forming protein complexes [21]. Thus, if the target genes of a TF have statistically significant overlap with a protein complex, this prevalence of protein-protein interaction may imply the trend that the TF-gene pairs are functional [31].

As proposed in [31], a protein complex is defined by two set of genes, the core genes and the neighbouring genes. Core genes are defined by the genes that are both assigned as the target genes and translated to gene products with physical protein-protein interaction. The set of neighbouring genes gathers the genes that are translated to the gene products having physical protein-protein interaction with the core genes. A protein complex is formed by the union of the core genes and the neighbouring genes.

Following the above definitions, a set of functional binding targets of a TF showed prevalence of protein-protein interactions if the proportion of the interacting proteins, or the core genes, in this set was significantly higher than the proportion of the protein complex within the whole genome. By defining the protein complex as described, we then performed the one-tailed Fisher exact test to test the protein complex overlap significance with FDR correction [33] and a threshold of *α *= 0.05.

Among the 62 common TFs between our results and the results of the LLS method, 49 TF functional binding target gene sets (79.0%) extracted by our method showed prevalence of protein-protein interaction while only 42 TF functional binding target gene sets (67.7%) extracted by the LLS method did. And among 46 TFs between our results and the results of the ECS method, 34 TF functional binding target gene sets (73.9%) extracted by our method showed prevalence of protein-protein interaction, comparing with only 23 TF functional binding target gene sets (50.0%) extracted by the ECS method did. For the 18 common TFs between our results and the results of the MA algorithm, 14 TF functional binding target gene sets (77.8%) extracted by our method showed prevalence of protein-protein interaction in comparison with only 12 TF functional binding target gene sets (66.7%) extracted by the MA algorithm did (Figure 5). In summary, our method can extract functional binding target genes of TFs with better protein functional cooperation than previous approaches can.

#### Expression coherence analysis

It has been shown that functionally relevant target genes of TFs tend to have similar mRNA expression profiles [34]. Using this notion, we calculated the Pearson correlation coefficients from the expression vectors between any two genes [18]. It has been pointed out that the TF-gene pairs are usually functional under different cellular conditions [10]. Hence, we collected 40 mRNA expression time series profiles under different conditions, as described in the Material section, and verified the expression coherence under these conditions. Since both positive correlation and negative correlation are both functionally relevant, we took the squares of the coefficients as our expression correlation measurement. Then under different conditions we performed the one-tailed rank sum test on the expression correlation coefficients to compare the expression coherence between two lists of functional binding target genes of TFs from different methods. We tested on the two different hypotheses: (1) the means of the square of correlation coefficients for the TF functional targets mined out in this study are *higher *than those generated by other methods (2) the means of the square of correlation coefficients for the TF functional targets mined out in this study are *lower *than those generated by other methods. Multiple hypotheses test correction was done by FDR correction and a threshold of *α *= 0.05 was adopted.

In different expression time series conditions, we first counted the percentage of the functional binding target sets of TFs with statistically higher expression coherence. Note that the percentages of more expression coherent functional binding target sets of TFs in two different methods may not add up to 100% since some of the TF functional binding gene sets may have statistically invariant average expression correlations between two methods. Then when one method gained more functional binding target sets of TFs than the other, we said that this method is more expression coherent than the other under this expression time series condition.

**Figure 6:**
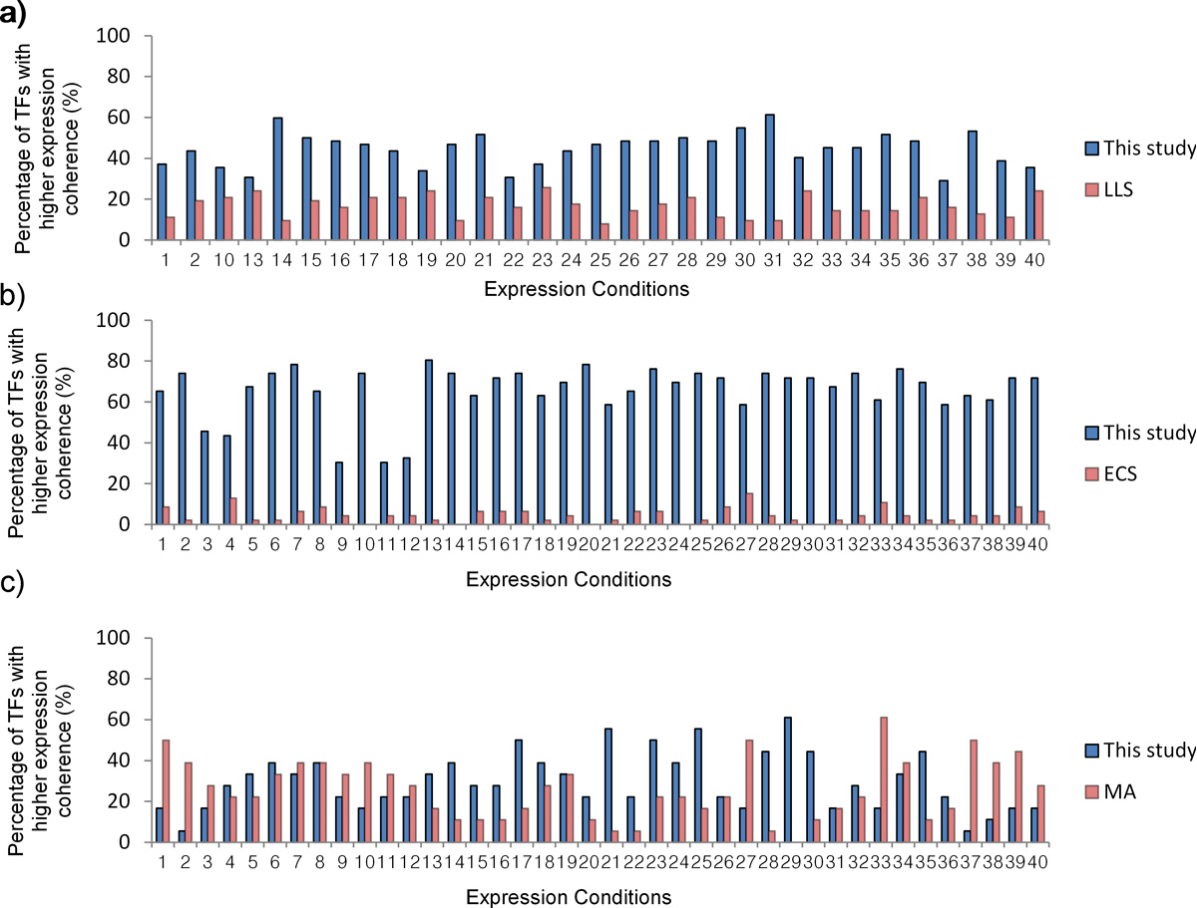
**Expression coherence validation**. Functional binding target genes of TFs identified by different methods were validated by expression coherence comparison under 40 different expression conditions. Note that the total numbers of TFs in each chart differ and correspond to the number of common TFs between those available from the method under comparison and from this study. And also note that the bars may not add up to 100 since some of the TF functional binding gene sets may have statistically invariant average expression correlations between this study and the method under comparison. a) Our results compared with results of the LLS method (62 common TFs). b) Our results compared with results of the ECS method (46 common TFs). c) Our results compared with results of the MA method (18 common TFs).

We compared our results with those of LLS, ECS, and MA methods for all 40 different expression profiles. Compared with the LLS method, our method conveyed better expression coherence under 31 different conditions. Our results were more correlated in the expression profiles than the results of the LLS method under most of the conditions. And our method showed more expression coherent pairs than the ECS method did under all 40 different conditions. Finally, compared with the MA algorithm, our method stood out under 21 conditions while the results of MA algorithm got better expression coherence under 15 conditions (Figure 6). Our methods were still more correlated in the expression profiles than the results of the MA algorithm. All in all, our method can extract functional TF-gene binding pairs with better expression coherence.

### Comparison with random assignments

To make statistical assessment of the results in this study, we made simulations against random assignments of functional/non-functional TF-gene pairs. In our study, we have shown that about 82% of the original TF-gene binding pairs suggests functionality. Hence we randomly removed 18% of the original binding targets from the 72 analysable TFs as the random assignment of non-functional TF-gene binding targets. We repeated this process for 50 times and gained 50 randomly assigned functional TF-gene binding pair lists. Then we performed the biological significance validation for the randomly assigned results as the stochastic lower limit of the performance of the validation methods.

After that, for the functional enrichment validation and the prevalence of protein-protein interaction validation, we used the left-tailed one sample student *t*-test to assess the significance of our result, compared to randomly generated TF-gene assignments for these 72 analysable TFs. The test was performed on the hypothesis that the average performance of the random results are statistically lower than the results in this study. As for the expression coherence validation, we performed the paired two sample *t*-test for our results and the random ones in every expression condition. In each condition-specific expression profile, we used the rank sum test as described earlier on the two stated hypotheses to compare the expression coherence between the result in this study and the randomly assigned TF-gene lists. The number of target gene sets satisfying the hypothesis of "results in this study is better than the random results" and the number of target sets satisfying the hypothesis of "results in this study is worse than the random results" for the comparison of our results to the 50 randomly assigned TF-gene lists formed the testing pairs. We said that our result is better than random assignments in the specific condition if we have the right-tailed *p*-value by the paired *t*-test on the 50 testing pairs below 0.05 in this condition.

As shown in Table 1 to Table 3 and Additional File 5, the overall performance of results in this study is statistically better than the 50 random TF-gene target lists (with *p*-value threshold of 0.05). For the 72 TFs, our results generated 62 functionally enriched functional binding target gene sets of TFs, compared to the performance of random assignments with mean and standard deviation equal to 44.8 and 3.53, respectively (one-tailed *p*-value = 2.76 × 10^-36^). For the validation of prevalence of protein-protein interaction, there were 53 functional binding target gene sets of TFs in this study showing prevalence of protein-protein interaction, while the random lists obtained a performance with mean and standard deviation of 17.68 and 3.78, respectively (one-tailed *p*-value = 7.04 × 10^-50^). Finally for the expression coherence validation, in 39 of the 40 different expression conditions our results were statistically more expression-coherent than the 50 random lists. Detail of the validation results can be found in Additional File 5 and Additional File 6. In summary, the results generated by our method are statistically meaningful and outperforms mere random assignments.

**Table 1:** Comparison of our results generated from the dataset of Harbison *et al*. to randomly assigned TF-gene pair lists for functional enrichment and prevalence of protein-protein interaction validation.

	**Harbison et al**.		
	**This study**	**Random result**	**One-tailed *p*-value**

Functional enrichment	62/72	44.8 ± 3.53	2.76E-36 (*t *= -34.41)
Prevalence of PPI	53/72	17.68 ± 3.78	7.04E-50 (*t *= -66.13)

**Table 2:** Comparison of our results generated from the dataset of Lee *et al. *to randomly assigned TF-gene pair lists for functional enrichment and prevalence of protein-protein interaction validation.

	**Lee *et al***.		
	**This study**	**Random result**	**One-tailed *p*-value**

Functional enrichment	42/46	31.84 ± 3.28	3.11E-27 (*t *= -21.87)
Prevalence of PPI	34/46	13.82 ± 3.21	1.48E-41 (*t *= -44.43)

**Table 3:** Number of conditions where our results are more statistically expression-coherent than random results are.

	**Harbison *et al***.	**Lee *et al***.
Expression coherent conditions	39	40

### Applicability to different datasets

To show that our method for identifying functional TF-gene binding pairs is not dataset-dependent, we also performed our method on the dataset provided by Lee *et al. *[2]. Since the original binding dataset used in the ECS method and MA algorithm was from the experimental analysis of Lee *et al*., we compared the biological relevance of this result with those generated by the ECS method and the MA algorithm. In applicable biological validations, similar conclusion also held for these comparisons (See Additional File 4).

For statistical assessment of the our results obtained by applying our method to the dataset of Lee *et al*., similar statistical significance also held (See Table 2 and Table 3). Detail of the validation results can be found in Additional File 5 and Additional File 6.

### Applicability to ChIP-seq datasets and the *E*. *coli *genome

The approach described in this study is not restricted to the Yeast genome or to merely ChIP-chip data. We further demonstrated that our method can applied to ChIP-seq binding datasets and to the *E. coli *genome data.

ChIP-seq provides a promising way for identification of transcription factor binding sites, but requires high quality of antibodies to the transcription factors. Thus the technique is still not scalable to the genome-wide scale of transcription factors [35]. While in yeast this is already done by ChIP-chip, no similar work has yet been done repeatedly for ChIP-seq. Hence we only showed the applicability of our method to the binding target of Ste12 identified by ChIP-seq. We adopted the ChIP-seq data for genome-wide Ste12 transcription factor binding sites from the work of Lenfrancois *et al. *[36]. In their work, the binding targets were manually curated and provided in the form of binding *p*-values. We took the binding targets of Ste12 with the *p*-value threshold of 0.05, as suggested in their analysis. A total of 926 targets of Ste12 were established from their experimental results. Ste12 is a transcription factor known to involve in mating and cell fusion [37]. Hence we tested the Gene Ontology enrichment of the original binding target list and the functional target list filtered by our method. Significantly enriched GO terms (FDR corrected *p*-value < 0.001) related to cell fusion (GO:0000747, conjugation with cellular fusion) and mating (GO:0019236, response to pheromone) were identified in the filtered functional TF-gene binding targets but not in the original target list (Table 4). This shows that our method can extract functional binding targets from the original ChIP-seq dataset.

**Table 4:** Cellular fusion and mating GO term enrichment for the original binding targets and filtered functional binding targets of Ste12.

	Original target list	Filtered functional targets
Cellular Fusion	33/926 (*p *= 0.00115)	9/48* (*p *= 7.36*E*10^-6^)
Response to Pheromone	31/926 (*p *= 0.00133)	7/48 * (*p *= 8.6*E*10^-4^)

(*indicates statistically enriched.)

**Figure 7:**
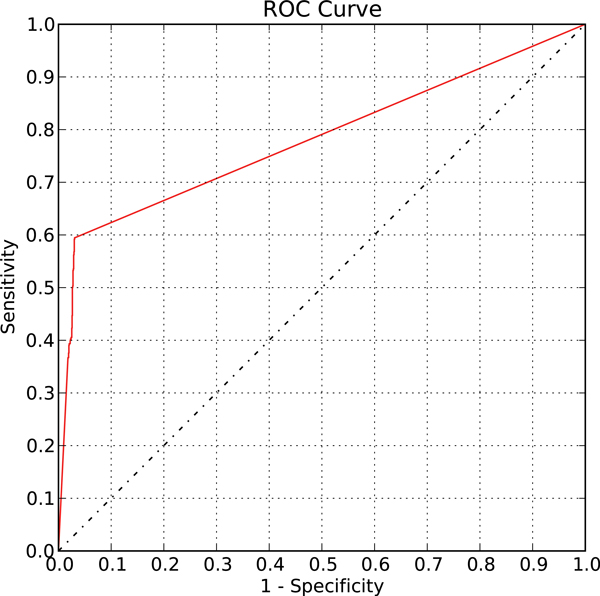
**The ROC curve of the validation on the ground truth set for E. coli**. When applied to the control set for *E. coli*, our method can distinguish functional binding targets from non-functional binding pairs, shown by the upper left corner trend on the ROC curve plot with AUC= 0.78. The ground truth set consists of 338 literature-curated functional TF-gene binding pairs as the positive control set and 1662 random TF-gene pairs as the negative control set.

We also demonstrated the applicability of our algorithm to the genome-wide data of *E. coli*. Since there is no other similar analysis for *E. coli*, we gathered a literature-proven benchmark functional TF-gene binding pair set for *E. coli *and performed our algorithm on this ground truth dataset. The benchmark set of 338 functional TF-gene binding pairs with at least three different experimental supports was collected from RegulonDB [38]. The negative control set was generated as described in the Method Section. We also collected 3990 TF-gene regulation pairs, which conveyed the same information as the TFKO data, from RegulonDB. Then we used these TF-gene regulation pairs to construct the *E. coli *regulatory relation network. Applying our method on the prepared control set, we can obtain the ROC curve by varying the RCSs. As shown in Figure 7, our method acted as a good classifier for discriminating functional binding pairs from non-functional binding pairs with AUC = 0.78. Hence our method can well-suited for the E. coli genome as well.

### Biological applicability of our method

We have listed the potential epistatic regulation cascade for every functional TF-gene binding pairs settled in this study (Additional File 2). To demonstrate the biological applicability of our method, we took the literature-proven functional TF-gene binding pair (Leu3p, *BAP2*) as an example. *BAP2 *is a gene encoding a permease in *Saccharomyces cerevisiae *for the uptake of branched-chain amino acids from media containing nitrogen source [39]. Deletion of *BAP2 *greatly reduced the up-take of leucine, isoleucine and valine. And Leu3p is a TF in yeast that regulates the transcription of a group of genes involved in leucine biosynthesis [40]. In the original binding dataset from the work of Harbison *et al*., the promoter region of *BAP2 *was found to be bound by Leu3p (with binding *p*-value of order 10^-7^). But there were no single TF knockout evidence showing the regulation of *BAP2 *by Leu3p. In our method, we found out that although there were no TFKO evidence for this TF-gene pair, we could find a regulation cascade (Leu3p → Msn4p → Rpn4p → Yap1p → Stp1p → BAP2) in the constructed regulation relation network through the TF Stp1p with RCS bigger than 1000. Hence we concluded that the ChIP positive (Leu3p, *BAP2*) has hypostatic regulation evidence and is a functional TF-gene binding pair.

The Leu3p binding site of *BAP2 *was established by computer assisted analysis [41]. In their work, Nielsen *et al. *also showed that mutating the Leu3p binding site reduced the transcription level of *BAP2 *on SC medium and concluded that Leup3 binding is required to obtain full BAP2 promoter activity. This matches our classification of the functional binding of Leup3 to *BAP2*. They also demonstrated that Stp1p can functionally bind to the promoter of *BAP2 *independently of the presence of the functional binding of Leu3p to *BAP2 *and are synergistic with Leu3p, suggesting the possible masking effect on the knockout event of Leu3p on *BAP2*.

## Conclusion

Inferring functional TF-gene binding pairs serves as the first step toward under-standing the regulatory pathways in cells. We have demonstrated that by integrating the ChIP-chip data with the TFKO data, our method can infer functional TF-gene binding pairs. And compared with three previous works, our method generated more biologically relevant results. Using our identified functional TF-gene binding pairs, it is possible to reconstruct a more reliable cellular transcriptional network, which will be helpful to unravel the unknown cellular mechanisms in future researches.

## List of abbreviations used

ChIP: chromatin immunoprecipitation; TF: transcription factor; TFKO data: transcription factor knockout data; PPI: protein-protein interaction; LLS: log likelihood score method; ECS: expression coherence score method.

## Competing interests

The authors declare that they have no competing interests.

## Authors' contributions

WSW conceived the research topic and provided essential guidance. THY developed the algorithms and wrote the manuscript. THY performed all the simulations and analysis. WSW proofread the final manuscript. Both authors have read and approved the final manuscript.

## Supplementary Material

Additional file 1 **Percentage of functional binding targets**. Additional file 1 contains the table showing the percentages of functional binding target genes of TFs with available TFKO data. In the table, the number of binding targets specified in the original binding data and the number of functional binding targets mined out by our method are shown. The percentage of functional target genes was calculated by the number of functional binding targets divided by the number of original binding targets. Click here for file

Additional file 2 **Functional TF-gene binding pairs**. Additional file 2 contains the table of functional TF-gene binding pairs mined out by our method. The potential epistatic regulation cascades were also listed in the table for possible subsequent analysis. Note that epistatic regulation cascades of path length one refer to the directly curated TFKO evidence. Click here for file

Additional file 3 **Biological significance validation on the results generated from the dataset of Harbison *et al***. Additional file 3 zipped the raw files for the three biological validations: 1) Prevalence of protein-protein interaction validation 2) Functional enrichment validation 3) Expression coherence validation. Detailed description of the raw files is written in the file 'ReadMe.doc' in Additional File 3. Click here for file

Additional file 4 **Supplementary validation for the biological significance on the results using the dataset generated by Lee *et al***. Additional file 4 contains Figure S1-S3 showing the biological significance validation on our results using the dataset generated by Lee *et al. *Figure S1 demonstrated the percentages of functional binding target genes of TFs with available TFKO data. Figure S2 showed the results of functional enrichment validation and prevalence of protein-protein interactions validation. Figure S3 showed the results of the expression coherence comparison. Click here for file

Additional file 5 **Summary of the comparison of our results to the 50 randomly assigned TF-gene lists**. Additional file 5 contains the table showing the detailed summary for Table 1 to Table 3, both for the results generated using the dataset of Harbison *et al. *and the results generated using the dataset of Lee *et al*.. Click here for file

Additional file 6 **Validation results of our results compared to 50 randomly assigned TF-gene lists**. Additional file 1 zipped the raw files for the three biological validations on the comparison of our results, both for the results generated using the dataset of Harbison *et al. *and the results generated using the dataset of Lee *et al*., to the 50 random TF-gene lists. Detailed description of the raw files is written in the file 'ReadMe.doc' in Additional File 6. Click here for file

## Declarations

The full funding for the publication fee came from Taiwan National Science Council and College of Electrical Engineering and Computer Science, National Cheng Kung University.

This article has been published as part of *BMC Systems Biology *Volume 7 Supplement 6, 2013: Selected articles from the 24th International Conference on Genome Informatics (GIW2013). The full contents of the supplement are available online at http://www.biomedcentral.com/bmcsystbiol/supplements/7/S6.
