# Supplementary material for: Inferring functional transcription factor-gene binding pairs by integrating transcription factor binding data with transcription factor knockout data
Source: BMC Syst Biol. 2013 Dec 13;7(Suppl 6):S13. doi: 10.1186/1752-0509-7-S6-S13 (PMC4029220; doi:10.1186/1752-0509-7-S6-S13)
Supplement: Additional file 4 — Supplementary validation for the biological significance on the results using the dataset generated by Lee et al. Additional file 4 contains Figure S1-S3 showing the biological significance validation on our results using the dataset generated by Lee et al. Figure S1 demonstrated the percentages of functional binding target genes of TFs with available TFKO data. Figure S2 showed the results of functional enrichment validation and prevalence of protein-protein interactions validation. Figure S3 showed the results of the expression coherence comparison. [file 1752-0509-7-S6-S13-S4.PDF]

## Supplementary Data

**Figure S1 - The percentage of functional binding targets of TFs extracted from the original ChIP-chip dataset**

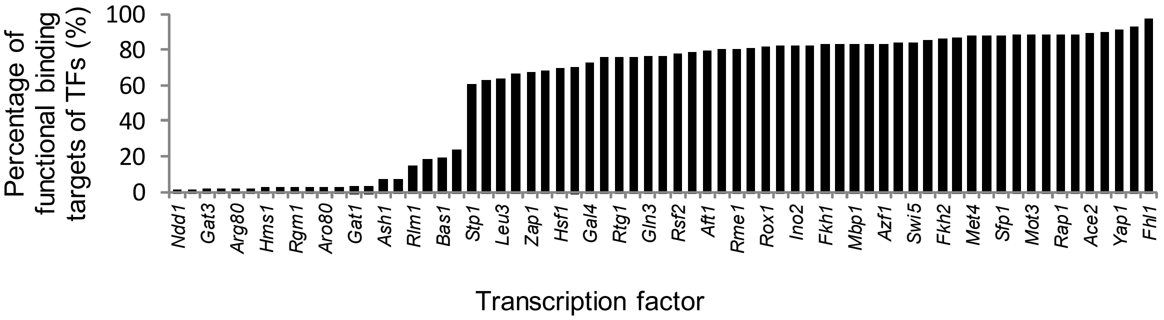


We generated another result by using Lee's data as the input ChIP positives. As described in the text, we used a 'jump' in the percentage plot to filter out the TFs that were lack of TFKO data. For the results generated from Lee's data, a 30% cut-off was adopted.

**Figure S2 - Functional enrichment and prevalence of protein-protein interactions validation**

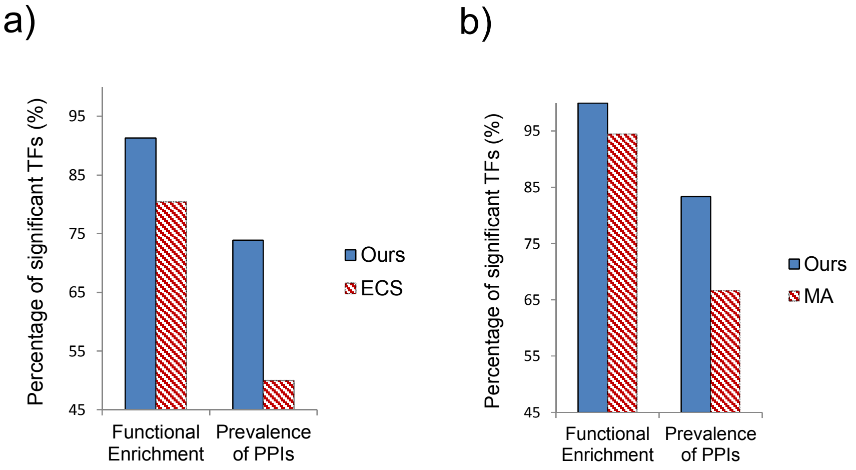


As described in the text, we compared our results from Lee's data to those of the ECS method and the MA method. a) Our results compared with results(42 functionally enriched TFs and 34 TFs with prevalent of PPIs) of the ECS method (37 functionally enriched TFs and 34 TFs with prevalent of PPIs in 46 common TFs). b) Our results(18 functionally enriched TFs and 15 TFs with prevalent of PPIs) compared with results of the MA method (17 functionally enriched TFs and 12 TFs with prevalent of PPIs in 18 common TFs).

**Figure S3 - Expression coherence comparison**

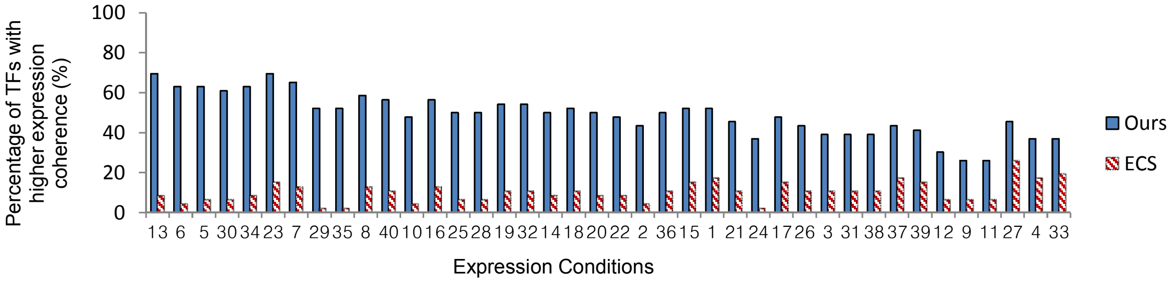


Functional binding target genes of TFs identified by different methods were validated by expression coherence comparison under 40 different expression conditions. Most of the mRNA expression datasets were used as the training data in the MA algorithm. Hence it is not suitable to conduct the expression coherence test for the MA method. In the plot are the comparison of our results compared with results of the ECS method (40 better expression coherent condition of our results in 46 common TFs).
