# Supplementary material for: Inferring functional transcription factor-gene binding pairs by integrating transcription factor binding data with transcription factor knockout data
Source: BMC Syst Biol. 2013 Dec 13;7(Suppl 6):S13. doi: 10.1186/1752-0509-7-S6-S13 (PMC4029220; doi:10.1186/1752-0509-7-S6-S13)
Supplement: Additional file 3 — Biological significance validation on the results generated from the dataset of Harbison et al. Additional file 3 zipped the raw files for the three biological validations: 1) Prevalence of protein-protein interaction validation 2) Functional enrichment validation 3) Expression coherence validation. Detailed description of the raw files is written in the file 'ReadMe.doc' in Additional File 3. [file 1752-0509-7-S6-S13-S3.zip › AddFile3/Supplementary Validation Results/ReadMe.doc]

This folder contains the raw files for the biological validations. There are three different types of validation:

1. PPI Prevalence Validation

2. Functional Enrichment Validation

3. Expression Coherence Validation

---------------------------------------------------------------------

1. Protein Cooperation Validation

In this folder, there are three files for the protein complex enrichment tests, compared with the LLS method, the ECS method and the MA algorithm, respectively.

----------------------------------------------------------------------

2. Functional Enrichment Validation

In this folder, there are three files for the functional enrichment terms found by GO TERM FINDER. One file contains the enriched GO terms for the comparison with the LLS method and the other file contains the enriched GO terms for the comparison with the ECS method. The last file contains the enriched GO terms for the comparison with the MA algorithm.

---------------------------------------------------------------------

3. Expression Coherence

In this folder, there are three summary files for the 40 different results and three folders containing the detailed statistical p values for tests under 40 different conditions.

Two different hypotheses are tested in comparison with two methods: a) Hypothesis1 with H0: our results are less coherent than the compared method b) Hypothesis2 with H0: the results of compared method are less coherent than our results.

Hence, when H0 were rejected, we concluded that our results out-performed the compared method under Hypothesis1. Similarly, we concluded that the compared method were better coherent under Hypothesis2 if H0 were rejected.
