# Supplementary material for: Inferring functional transcription factor-gene binding pairs by integrating transcription factor binding data with transcription factor knockout data
Source: BMC Syst Biol. 2013 Dec 13;7(Suppl 6):S13. doi: 10.1186/1752-0509-7-S6-S13 (PMC4029220; doi:10.1186/1752-0509-7-S6-S13)
Supplement: Additional file 6 — Validation results of our results compared to 50 randomly assigned TF-gene lists. Additional file 1 zipped the raw files for the three biological validations on the comparison of our results, both for the results generated using the dataset of Harbison et al. and the results generated using the dataset of Lee et al., to the 50 random TF-gene lists. Detailed description of the raw files is written in the file 'ReadMe.doc' in Additional File 6. [file 1752-0509-7-S6-S13-S6.zip › AddFile6/Supplementary Random Results/ReadMe.doc]

This folder contains the raw files for the biological validations between results generated by our method and random assignments. There are three different types of validation:

1. PPI Prevalence Validation

2. Functional Enrichment Validation

3. Expression Coherence Validation

---------------------------------------------------------------------

1. Protein Cooperation Validation

File name convention:

Protein_Cooperation_[original dataset]_random_[random number].txt

The file contains the comparison results between our results and the specified random list.

----------------------------------------------------------------------

2. Functional Enrichment Validation

File name convention:

( [our results] vs_random_[original dataset]_[random_number] )_[File of interest] _GO_webresult_detail.txt

The file contains the detailed significant GO terms of our results or the specified random list.

---------------------------------------------------------------------

3. Expression Coherence

File name convention:

expression_coherence_[Hypothesis type]_random_[original ChIP dataset]_[random_number]_[expression profile condition].txt

Two different hypotheses are tested in comparison with two methods: a) Hypothesis1 with H0: our results are less coherent than the compared method b) Hypothesis2 with H0: the results of compared method are less coherent than our results.

Hence, when H0 were rejected, we concluded that our results out-performed the compared method under Hypothesis1. Similarly, we concluded that the compared method were better coherent under Hypothesis2 if H0 were rejected.
